# Phenotypic characterization and quality of life of Labradoodles with idiopathic epilepsy and epilepsy of unknown cause

**DOI:** 10.3389/fvets.2024.1459260

**Published:** 2024-10-16

**Authors:** Paul J. J. Mandigers, Simone E. Meijs, Marta Plonek, Koen M. Santifort, M. Montserrat Diaz Espineira

**Affiliations:** ^1^Expertise Centre of Genetics, Department of Clinical Sciences, Faculty of Veterinary Medicine, Utrecht University, Utrecht, Netherlands; ^2^Evidensia Referral Hospital, Arnhem, Netherlands

**Keywords:** designer breed, dog, pedigree-dog, impact, owner, treatment

## Abstract

**Introduction:**

Idiopathic epilepsy (IE) is a common chronic neurological disorder in dogs. Breed specific knowledge on semiology, age of onset (AoO), seizure frequency, response to treatment, and Quality of Life (QoL) scores for both dog and owner are important. The Labradoodle is originally a cross between the Labrador Retriever and Standard Poodle. The aim of this study was to describe in detail the epilepsy, that is recognized in this designer breed.

**Method:**

An online survey was distributed through several platforms to owners of Labradoodles. Only Labradoodles, either registered as such or bred as a Labradoodle were included. To classify the Labradoodles in a TIER level of confidence the criteria of the International Veterinary Task Force were used.

**Results:**

Forty labradoodles were included. Thirty-three Labradoodles were classified as IE or suspected IE cases and appeared to have an AoO of 2.3 ± 1.3 years, and a seizure frequency of 5.4 ± 6.5 seizures/year (range 0–25). Seven dogs were classified as EUC, two dogs started before 6 months of age, and five dogs had their first seizures at 8.4 ± 1.2 years of age. The seizure frequency for all EUC dogs was 5.0 ± 3.0 seizures/year (range 0–11). Stress, for both the (suspected) IE and EUC cases, was the most frequently reported seizure precipitating factor (95% of the dogs). AoO and seizure frequency did not seem to be influenced by type of housing, type of owner, or the dog’s character as interpreted by the owner. Cluster seizures (CS) were seen in 33% and status epilepticus (SE) in 10% of the included Labradoodles. Many dogs did not need treatment (38%) or only one anti-seizure medication (43%), in which case they tended to respond favorably to treatment. QoL scores were high for both dog (8.3 ± 1.8) and owner (7.3 ± 2.2).

**Discussion:**

Compared to the progenitor breeds, epilepsy of Labradoodles is phenotypically like that of the Standard Poodle, except for the existence CS and SE in the Labradoodle. The epilepsy seen in this designer breed has a favorable outcome compared to several other breeds.

## Introduction

Idiopathic epilepsy (IE) is a common neurological disorder in dogs (1, 2) and has been described in detail for various breeds, such as the Labrador Retriever (3), Golden Retriever (4), Italian Spinone (5), Irish Wolfhound (6), Vizsla (7), Border Collie (8, 9), Australian Shepherd (10), Rottweiler (11), Irish Setter (12), Belgian Shepherd (13), and Great Swiss (14). In many purebred dogs, IE is believed to be genetic and a complete overview has been provided in 2015 by the International Veterinary Epilepsy Taskforce (IVETF) (15) and in 2022 by Fischer (16). IE is subdivided into three groups: (1) genetic epilepsy, (2) suspected genetic epilepsy, and (3) epilepsy of unknown cause (1). The latter means epilepsy in which the nature of the underlying cause is yet unknown and with no indication of structural epilepsy (1). To classify a dog as suffering from IE the history should reveal two or more unprovoked epileptic seizures occurring at least 24 h apart, age at epileptic seizure onset of between 6 months and 6 years, an unremarkable inter-ictal physical and neurological examination, and no clinically significant abnormalities on minimum data base blood tests and urinalysis (17).

Epilepsy is a debilitating disorder that impacts the life of both dogs and their owners, and its clinical phenotype varies per breed (18). A number of QoL studies investigating this impact in different breeds have been published in the recent years (9, 19–21) and clear differences are seen for the investigated breeds. For instance, the QoL is low for both owner and dog, the response to treatment is poor, and the life expectancy is short for the Border Collie and Australian Shepherd (9, 10, 18) compared to the Labrador Retriever and Golden Retriever with much higher QoL scores and better outcome (18).

But IE does not just affect purebred dogs and is also seen in crossbreeds and village dogs (18, 22). Although pedigree dogs continue to be popular, there is a growing interest in designer breeds such as the Pomchi (Pomeranian × Chihuahua), Cockapoo (Cocker × Poodle), Puggle (Pug × Beagle), Maltipoo (Malteser × Poodle), Pomsky (Pomeranian × Husky), Goldendoodle (Golden Retriever × Poodle), and Labradoodle (Labrador Retriever x Poodle).^1^ Of these, the Labradoodle seems to be exceptionally popular (23, 24). A designer breed results from crosses between existing breeds that aim to enrich the offspring with desired traits and characteristics of the progenitor breeds (23–25). The Labradoodle originated in 1989 and was initially a cross between the Labrador Retriever and Standard Poodle with the aim to breed a low-shedding service dog. With some additional crosses with the Irish Water Spaniel, Curly Coated Retriever, and the English and American Cocker Spaniel, this finally resulted in the Australian Labradoodle (25, 26). In several countries, there are Labradoodle breed clubs that register dogs with several generations of Labradoodle ancestry and provide owners a pedigree, including a parental DNA check.^2^^,^^3^ Although not recognized by the Fédération Cynologique Internationale (FCI) or the American Kennel Club (AKC), the Labradoodle is bred, controlled, and registered like any ‘official’ breed which makes it difficult to deny that it is a breed (see footnote 2 and 3). Many owners believe that designer breeds are genetically healthier because they are cross-bred (24, 25, 27). However, this is in contrast with reports describing genetic diseases like Addison’s disease, progressive retinal atrophy, hip dysplasia, and elbow dysplasia in both the progenitor breeds as well as the Labradoodle (23, 26, 28–30). And a recent study analyzed data of 583 Labradoodles and compared it with two of the progenitors, the Labrador Retriever and Standard Poodle. The findings of the authors are, in contrast with the general perception of Labradoodle owners, that the Labradoodle is not healthier than the progenitor breeds (25). Compared to the Poodle, the Labradoodle did not differ in their odds for 50 out of 57 disorders and compared with the Labrador Retriever for 52 out of 57 disorders (25). Epilepsy has been described in both the Labrador Retriever (18, 31–33) and the Standard Poodle (34). It has been mentioned to occur in the Labradoodle in two different studies not specifically addressing this breed (18, 31). In the recent study of Bryson et al. (25), survey based, the reported frequency of epilepsy was 1.7% for the Poodle, 0.9% for the Labrador Retriever and 1.4% for the Labradoodle. However, details on, for instance, AoO, type of seizures, phenotypic characterization, treatment response, and QoL of both dog and owner were not described in any of these studies.

The objective of this study is to describe the phenotypic characterization of epilepsy (suspected IE, IE, and EUC), QoL for both owner and dog, and response to treatment for the Labradoodle. Furthermore, we aimed to investigate the influence of other variable including housing, character of dog (anxious versus calm and steady), and type of owner (calm versus stressed) on AoO and seizure frequency.

## Materials and methods

### Data collection

A web-based questionnaire, created with Qualtrics^®^ survey software,^4^ was distributed in the Netherlands via Facebook, LinkedIn, three online canine epilepsy fora, two online Labradoodle fora, a Dutch newspaper, and direct emailing of owners and veterinarians in the Netherlands, to reach owners of Labradoodles with epilepsy. The questionnaire was made available in Dutch and English (Appendix 1, 2) and was based on those used in previous studies by our group (9, 18). It was open to responses from January to July 2023. All questions were accompanied by an explanation of the used medical terms, a web page was available with an explanation in Dutch and English, and videos of a generalized tonic–clonic epileptic seizures (GTCS), focal epileptic seizures (FS), and paroxysmal dyskinesia (PD) were accessible on YouTube to watch. Participating owners were asked to contact us if they had any questions. If the provided answers were unclear, we contacted the owners for clarification. All owners consented to participating in the study at the beginning of the questionnaire. They were informed that we would not store their IP addresses or disclose any other information that could be used for identification. They were asked to fill in the questionnaire only once unless they had more than one Labradoodle with epilepsy.

### Questionnaire

The questionnaire consisted of 127 questions and was divided into several sections:

1. To gather information about the owner and environment multiple choice questions (MCQs) about the character of the owner(s), living environment and, if applicable, other pets.
2. MCQs and open questions about the type of doodle and registration, date of birth, sex and neuter status, nutrition, vaccination status, anthelmintic and anti-ectoparasitic drug use, and other possible health problems.
3. Living conditions of dog and owner: MCQs about the character of the dog and living conditions.
4. MCQs and open questions about the first and most recent seizures, frequency, type, cause and the diagnostics performed by the practitioner and/or specialist.
5. Additional questions: MCQs, open, and rating scale questions about the number of seizures in the first and last year, the severity, possible triggers, recognition, length and signs of the pre-ictal phase, the length of the seizures.
6. MCQs about the semiology of the seizure(s), differences in presentation.
7. Questions about the post-ictal phase. MCQs and open questions about the recognition, length, and clinical signs.
8. MCQs and open questions about the diagnostics performed and to extract information on other possible health problems.
9. Sex-specific questions (MCQs and open questions) about the heat cycle (for female dogs), sexual behavior and possible offspring.
10. Numbers, seriousness and control: MCQs, open, and rating scale questions about the number of seizures, cluster seizures (CS), status epilepsy (SE), severity, and veterinary visits.
11. Medication: MCQs and open questions about anti-seizure medication (ASM), emergency medication, side effects, and alternative treatment.
12. Quality of life (QoL) scores: ten rating scale questions about the dog’s and owner’s QoL, regarding worrying about the frequency, acceptability of severity, fear about leaving the dog alone, whether caring is worth it, administration of medication, acceptability of side-effects, veterinary visits and costs.

### Inclusion criteria

All types of Labradoodles were included, provided it was either registered as a Labradoodle or bred as a Labradoodle. Only dogs with at least 2 epileptic seizures were included. The classification and diagnostic criteria described by the IVETF were applied (1, 17). Hence dogs could only be included if there was a fitting semiology, history, unremarkable physical and neurological examination without indication for a reactive or structural epilepsy. Diagnostic level of confidence was classified as IE TIER I if the AoO was between 6 months and 6 years of age and additional blood-, and urine tests revealed no abnormalities (1, 17). It was labeled TIER II if a magnetic resonance imaging (MRI) of the brain, cerebrospinal fluid (CSF) analysis, and fasting pre- and postprandial bile acid measurement were normal (17). A dog was classified, in this study, as EUC if the dog was either younger than 6 months or older than 6 years at the time of seizure onset and reactive as well as structural cause were excluded (TIER II level of confidence) (1). If the semiology, history and physical examination was unremarkable, the dog had been suffering from seizures over a period of at least 1 year without indication for a reactive or structural epilepsy, but the TIER I diagnostic criteria were not met (for instance a blood-examination or urine analysis was missing) the dogs was classified as suspected IE.

### Exclusion criteria

All responses that lacked essential information were excluded. The following information was considered essential: Labradoodle type, sex and neuter status, whether the dog was alive or deceased, cause, type, and total amount of seizures, and ASM usage. Furthermore, all dogs with an abnormal physical and/or neurological examination, and/or abnormal MRI finding, and/or a history that suggested that it was not an IE or EUC were excluded.

### Data analysis

The collected answers were imported into Microsoft^®^ Excel 2021 to manually determine the reliability of responses and to check for compliance with the inclusion and exclusion criteria. IBM^®^ SPSS Statistics (Version 29) was used to analyze the data. First, a descriptive analysis was performed and are reported as mean ± standard deviation. Only for Labradoodles diagnosed suffering from epilepsy for at each 1 year, the average, range, total number of seizures, clusters and status epilepticus will be calculated. For normal distributed data, an independent-samples T-test was used to test for significant differences between groups. Significant correlation between two nominal variables was tested with a chi-square test if the values were ≥ 5, and otherwise a Fisher’s exact test was used. AoO, seizure frequency, duration of seizures, QoL scores are reported in mean ± sd and for frequency also the range. For all these tests, results were considered statistically significant if *p* < 0.05.

## Results

### Dogs

There were 222 responses, of which 167 were excluded due to incomplete data. One response was excluded for the dog was a Goldendoodle. Two responses were excluded as it was a structural epilepsy: one dog with polymicrogyria and one with a brain tumor (both MRI diagnosed). Eleven responses concerned dogs with a paroxysmal dyskinesia (35) and one had an idiopathic head tremor (36). In total, the data of 40 labradoodles with IE or EUC could be analyzed.

Thirty-two out 40 (80%) were Labradoodles with an official registration as Australian Labradoodle, 8 (20%) were Labradoodles without an official registration. One response came from Finland, one from Sweden, one from the USA, one from Canada, and the other 36 from the Netherlands. The number of males (*n* = 27; 68%) was statistically significantly higher (*p* < 0.01) than females (*n* = 13; 32%). All females had been neutered. Seventy-five percent of the males were neutered and 25% not. The majority of the dogs (*n* = 27; 68%) were neutered prior to their first seizure. Six males were castrated after their first seizure. There was no difference for AoO or seizure frequency between these two groups nor did the owners report any effect on seizure frequency after the castration.

At the time of the questionnaire, 38 dogs were still alive, two were dead. One dog was diagnosed as IE and one EUC, both dogs died at an older aged.

### Seizure types, age of onset (AoO), and seizure frequency

Twenty-five dogs had only GTCS, 12 had a combination of GTCS and FS (independently occurring of each other), three had only FS. There was no correlation with sex. Twenty-eight dogs were classified as IE, seven as EUC (with a TIER II level of confidence) and five as suspected IE as a blood examination was missing in these five dogs. At the time of inclusion, these five dogs had been suffering from GTCS over a period of 1 year and are therefore classified as suspected IE. The average age of onset was 2.3 ± 1.3 years for the dogs with IE. Two dogs classified as EUC were 3.5 months old when the seizures were first seen (two males, one GTCS and one GTCS + FS). The other five dogs with EUC had their first seizures at 8.4 ± 1.2 years of age (two males, three females; four GTCS and one FS). The total number of seizures, for the labradoodles diagnosed with epilepsy for a period of at least 1 year, was 5 ± 6.5 seizures/year and ranged from 2 to 90 seizures. If only the last year was evaluated, seizure frequency was 5.4 ± 6.5 seizures/year (mean ± sd). The range for dogs treated with ASM was zero to 25 with a median of three seizures/year and for dogs not treated with an ASM was zero to eight with a median of three seizures/year. The severity of the GTCS was scored on average with 6.9 ± 2.0 with zero meaning not severe and 10 very severe. The severity of the FS scored 3.7 ± 3.2.

### Duration and semiology of the seizures

On average the duration of the seizures was 3.1 ± 1.4 min. Next to the classical signs, such as lying flat on their side (80%), tonic–clonic movements of the limbs (95%), and salivation (85%), some additional signs were noted by the owners. Most dogs (90%) fell on the floor after which the seizure started. Urination was seen in 75%, defecation in 43% of the dogs. Clear loss of consciousness was reported by owners in 63% of the dogs. A same number showed clear chewing movements. Forty % of the owners observed dilated pupils, loss of vision (30%), turning of the head (55%), and vocalization (20%). Lateralisation was visible in 15% of the dogs. None of the owners was able to distract the dog or shorten the seizure. There was no difference in semiology noted for the dogs with suspected IE, IE or EUC.

### Pre- and post-ictal phase

Seventy percent of the owners (*n* = 28) was not able to predict a coming seizure, but 5 owners were always able to predict a coming seizure and 7 occasionally. The pre-ictal signs could vary from 1 min to 2 h. Pre-ictal signs were restlessness (30%), vomiting prior to the seizure (22%), attention seeking (15%), drooling (7%), and nausea (5%). Around 80% of the dogs had the seizure while sleeping, just after waking up, or the dog was resting but awake. Three owners reported to have seen the seizures outside. During the seizure, 85% (*n* = 32) of the owners could not contact their dog. The other owners responded they thought the dog was aware of them. However, the dog was not responsive.

### Treating veterinarian

Twenty-four dogs were regularly seen by the owner’s local veterinary practitioner, 12 on a regular base by a veterinary specialist or resident in neurology (PM, MP, KS, or MD), and 4 dogs were only seen by a veterinarian if needed.

### Diagnostic tests

A physical and neurological examination revealed no abnormalities in any of the 40 dogs. Urine analysis was performed in all dogs and was found to be unremarkable. Blood tests (including hematology and biochemistry) were performed in 35 dogs, MRI in 7, an electrocardiogram in 2, and a cardiac ultrasound in 1 dog. No abnormalities were observed in any of the dogs. The five dogs in which a blood examination was lacking were described by the owners as normal cheerful dogs. All five dogs had been suffering from GTCS for at least 1 year. There was no statistically significant difference for any of the results between the suspected IE, IE or EUC cases. Five owners reported that their dog also had clinical signs suggestive for atopy, two for gastro-intestinal problems, one was diagnosed previously with Addison’s disease, and one was previously diagnosed with hypothyroidism. These co-morbidities were seen in all groups (suspected IE, IE and EUC).

### Cluster seizures and status epilepticus

Thirteen dogs (33%) suffered from CS, the other 27 dogs (67%) did not. Three dogs had 1 cluster per year, two dogs two cluster per year, one dog three, one dog 7, and one dog endured 16 clusters the last year. SE was seen in four dogs (10%). These dogs also suffered from CS. One dog had endured 4 status episodes in 1 year, the other 3 only one in a year.

### Medication, supplements, and side effects

Fifteen dogs (38%) did not receive any ASM. These dogs had a mean frequency of 3.6 ± 2.3 seizures per year. The other 25 dogs (62%) were administered either one ASM or a combination of ASM. Eleven dogs were treated with only phenobarbital, five dogs with only imepitoin, one with only levetiracetam. One dog was treated with a combination of phenobarbital and imepitoin, four with a combination of phenobarbital with levetiracetam, one with a combination of potassium bromide and gabapentin. These dogs had a mean frequency of 6.5 ± 8. Two dogs with a high seizure frequency and cluster seizures/status epilepticus were treated with a combination of phenobarbital, potassium bromide, and levetiracetam. One dog, not receiving any ASM, was administered cannabidiol (CBD) oil. One owner added medium chain triglyceride (MCT) oil to their dog’s diet.

Twelve of 25 dogs (48%) that received ASM did not show any side effects. Side effects were reported in 13 of 25 dogs (52%) receiving an ASM. The most frequent side effects were more sleeping (*n* = 9), increased appetite (*n* = 9), fatigue (*n* = 7), paresis (*n* = 6), incoordination (*n* = 4), polydipsia (*n* = 4), and weight gain (*n* = 3). The side effects were predominantly seen in the dogs treated with phenobarbital (*n* = 8) or a combination of 2 or more ASM (*n* = 5). The exception were dogs treated with imepitoin as a single ASM. Side effects were not reported for those dogs (*n* = 5).

In our study group, 38% of the dogs did not receive an ASM, and another 43% just one. Side effects were reported in 20% of the dogs, but this was not considered to be a problem. Giving medication to their dog and going to the veterinarian was not seen as a major issue nor was this the case for the financial costs.

### Emergency medication

Twenty-one owners (53%) did not use any emergency medication as it was not deemed necessary. Three used a nasal midazolam spray (7.5%), 14 rectal diazepam (35%), and three levetiracetam (7.5%). The latter was the so-called pulse therapy with a high dose the first day the seizures were seen and tapering it over a period of days (37).

### Owner, housing, and character of the labradoodle

The majority of the dogs were kept by a couple (*n* = 19), or a couple with children (*n* = 18). Most owners indicated that the dog stayed in a quiet, not too busy setting (*n* = 36), described themselves as stable person (*n* = 36) and the same applied for their partner/family (*n* = 34). All dogs were kept in-house and were walked on a regular base. Twenty-two owners also kept other animals [dog(s), cat(s), rabbit(s)] and in 4 situations the relation between the other animals was sometimes troublesome. These dogs did not differ in any way compared to the others. They had no earlier AoO, more seizures, or needed (more) ASM. Twenty-eight Labradoodles were described by their owners as vivid, cheerful with a stable character, and 11 were described as a nervous, anxious dog. The average number of seizures for the stable doodles, with IE, was 5.9 ± 7.2 seizures/year and the nervous Labradoodles with IE, 7.3 ± 9.8 seizures/year. However, this was statistically not significantly different (*p* = 0.231). The AoO of stable Labradoodles with IE was 2.2 ± 1.4 years and for the nervous doodles with IE 2.4 ± 0.7 years. Again, this was statistically not significantly different (*p* = 0.242).

### Vaccination, deworming, antiflea/tick treatment, and food

Most dogs had already received their first vaccination (*n* = 33), deworming (*n* = 32), and antiflea/tick treatment (*n* = 32) long before their first seizure. Most owners switched the food brand several times (*n* = 33). There was no correlation between any of these treatments, food changes, and the AoO and/or number of seizures.

### Seizure precipitating factors

All owners recognized seizure precipitating factors (SPF). Stress was the most frequently reported SPF, noted for 95% of the dogs. Visits to the veterinarian, trimmer, treatments for different reasons were reported preceding a seizure in three dogs. Other SPF were sexual arousal (*n* = 1), weather (*n* = 3), visitors at home (*n* = 2), season of the year (*n* = 2), and sounds (*n* = 1).

Thirty-nine owners observed a post-ictal phase. The time varied from 1 min to 2 days with a median of 25 min. The most frequent signs were fatigue (*n* = 31; 78%), pacing (*n* = 18; 45%), wanting to drink (*n* = 7;18%), wanting to eat (*n* = 6; 15%), wanting to rest (*n* = 8; 20%), aggression (*n* = 7; 18%), impaired vision/apparent blindness (*n* = 6; 15%), attention seeking (*n* = 5; 13%), wanting to go outside (*n* = 4; 10%), ataxia (*n* = 3; 8%), anxiousness (*n* = 2; 5%), and vomiting (*n* = 2; 5%). Only one owner, who’s dog had GTCS, could not observe a post-ictal phase.

Nineteen of the 40 owners (47.5%) reported to find the ictal phase the most distressing, while thirteen (32.5%) found the post-ictal phase most distressing. Four owners found both equally distressing (10%). Four did not express their opinion (10%).

### QoL and other scores

The QoL score (where 10 was optimal) for the dog was scored by 38 owners with an average of 8.3 ± 1.8. They scored their own QoL with 7.3 ± 2.2. Taking care of their dog was scored with an 8.8 ± 2.3. The owners scored their general concerns with a 5.2 ± 3.7 (0 = no worries, 10 = severe). A similar score was seen for ‘leaving their dog alone at home’: 4.2 ± 3.7. As for giving the medication on a daily basis, the score was 2.2 ± 3.4, for the side effects the score was 2.2 ± 3.4, and for visits to the veterinarian 2.5 ± 3.2. The burden of the financial costs was scored with a 5.1 ± 3.5 (0 = a huge problem, 10 = no problem at all). Owners were also asked to say what frequency they found acceptable. Nine owners hoped for less than one seizure per 3 months, 21 owners a maximum of one seizure per 6 months.

### Labradoodle genealogy

Although 32 Labradoodles had an official registration with a pedigree, it was not possible to establish a common pedigree. Almost none of the included dogs shared a common ancestry. However, seven owners indicated that their Labradoodle was related to another epileptic Labradoodle (first-degree relative, second-degree relative, *etcetera*).

## Discussion

This is the first study describing the semiology, seizure frequency, severity, response to treatment, and QoL for dog and owner in the Labradoodle. Although three earlier studies mentioned that Labradoodles could suffer from epilepsy, detailed information was lacking (18, 25, 31).

Labradoodles have the same type of seizures (CTCS and FS) with a comparable semiology as several other breeds (16, 18). In this study, we found a male predisposition for suspected IE/IE/EUC in Labradoodles. Similar observations, although not statistically significant, have been reported for the Border Collie (8, 9), Australian Shepherd (10), Belgian Shepherds (13), Bernese Mountain dog (38), and several other breeds (15). The AoO of IE (2.3 ± 1.3 years) is comparable with the published AoO of two prior studies on IE of the Labrador Retriever; a Swiss population had an average AoO of 2.3 to 2.8 years (3) and a Dutch/Belgium population had an average AoO of 3.1 ± 2.3 years (18). Both studies reported dogs with GTCS, a combination of GTCS and FS, and some dogs only having FS (3, 18). A similar result has been published for the Standard Poodles, although the authors reported a median of 3.7 years with a range of 6 months to 7 years (34). In dogs of that breed, a combination of GTCS and FS, and only FS were seen as well.

In our study, the seizure frequency for the Labradoodle (5.4 ± 6.4/year) was slightly lower than the reported frequency for the Labrador Retriever (7 ± 13/year) (18) but remarkably higher than for the Standard Poodles with a much lower frequency (34). Although an exact frequency was not reported in the study on Standard Poodles, 30/90 dogs had only shown one seizure and a smaller number only two or three seizures during the complete evaluation period of several years (34). Interestingly, only 3/90 Poodles received treatment (34). In both the study of Heynolds et al. (3) and Hamers et al. (18), all included Labradors Retrievers received an ASM. In our study group of 40 Labradoodles, 38% did not receive an ASM and 43% only one ASM. Cluster seizures or status epilepticus were not reported for the Poodle (34). The number of clusters for the Labradoodles is slightly higher compared with the number published for Labrador Retrievers but the number of SE events is comparable (18). The most important phenotypic characteristics for the Labradoodle, Labrador Retriever and Standard Poodle is summarized in Table 1.

**Table 1:** Comparison of the Labradoodle with the two most important founder breeds.

	Labradoodle	Labrador retriever	Standard poodle (33)
AoO	2.3 ± 1.3 years	2.3 ± 2.8 years (3) 3.1 ± 2.3 (17)	Medium 3.7 years (Range 0.6–7 yrs)
Seizure frequency	5.4 ± 6.5/year	7 ± 13/year (17)	Low
Seizure duration	3.1 ± 1.4 min	–	–
Clusters	33%	11% (17)	None
Status	10%	11% (17)	None
QoL dog	8.3 ± 1.3	7.7 ± 2.3 (17)	–
QoL owner	7.3 ± 2.2	8.6 ± 3.0 (17)	–
Severity GTCS	6.9 ± 2	6.1 ± 2.6 (17)	–
Severity FS	3.7 ± 3.2	3.6 ± 2.2 (17)	–

Compared to the Labradoodle and Labrador Retriever, the Standard Poodle has a favorable phenotype. The study of Licht et al. (33) did not provide the exact seizure frequency but based on the numbers mentioned it was very low. The figures from Hamers et al. (17) for the Labrador Retriever are comparable with the findings of this study for the Labradoodle although the Labradoodle has more clusters and a slightly lower QoL score for the owner, but a higher QoL score for the dog.

In this study, we also looked at the effect of the character of the dog, the type of owner, and housing as this may also have an influence of the expression of IE/EUC. Earlier, our group reported SPF in a small number of Border Collies with IE (39). However, we could not observe any direct SPF in this study as owners reported that the environment of the dogs was calm and stable. However, stress prior to seizure onset was reported by 95% of the Labradoodle owners. We found two groups of Labradoodle character types reported: ‘stable’ and ‘nervous.’ No statistically significant differences were found between these groups for AoO or seizure frequency.

In the Netherlands, Labradoodles are often neutered in their first year. Therefore, we were unable to assess an effect of neutering on the AoO and/or seizure frequency. In contrast to what we reported in the Border Collie (9), almost all Labradoodles were vaccinated, dewormed, and treated against fleas and tick prior to their first seizure. A large number of dogs had not received vaccination, deworming and flea and tick treatment prior to their first seizure. It is unclear why this difference exists.

The QoL of Labradoodles with (suspected) IE/EUC in this study was scored an 8.3 ± 1.3 which is comparable to results of earlier studies on the Chihuahua, Dachshund, and Golden Retriever (18). This is clearly in contrast with the QoL score of breeds such as the Border Collie and Australian Shepherd who scored around 6.4 ± 2.9 (18).

The Labradoodle is a crossbreed of Labrador Retriever and Poodle, as well as Irish Water Spaniel, Curly Coated Retriever, and the English Cocker Spaniel and American Cocker Spaniel. Based on a genetic study, the Poodle genome dominates (26). Apparently, this is in favor of the epilepsy we see in this designer cross. Although higher percentages of Labradoodles had CS and SE compared to the Standard Poodles, both dog and owner have a high QoL score, with 38% not needing treatment, and if treatment was needed, acceptable results could be reached in most dogs.

This study has limitations. First, despite our efforts to collect data on a large number of Labradoodles suffering from epilepsy, only 40 out 222 questionnaires could be included. Second, most dogs, but not all, had a TIER I level of confidence. A minimal work-up should entail at least a physical examination, neurological examination, blood and urine analysis. In five dogs a blood examination was lacking. But given the period these dogs were suffering from their seizures, in all five cases over a year, it is less likely to be a reactive epilepsy. Ideally, all dogs would have reached a TIER II level of confidence but both financial constraints as well as owner reluctance to consent to procedures that carry some risk such as CSF collection resulted in a low number of cases reaching TIER II. Although almost all Labradoodles showed clear GTCS, electroencephalographic confirmation would give a higher confidence level of the diagnosis (TIER III) (40). Third, it was not possible to establish the prevalence of IE/EUC in this designer breed. The exact number of Labradoodles bred is unknown which makes it impossible to establish the prevalence. Four, a pedigree analysis was, although several dogs had a pedigree, not possible as almost none of the included dogs shared a common ancestry.

IE is suspected to be (partly) genetic in most breeds (15, 16). In several breeds, IE is seen as a major welfare diminishing disease requiring a specific breeding strategy to reduce the prevalence. Although our results suggest that IE seen in the Labradoodle designer breed has a more favorable clinical phenotype than other pedigree breeds, it too is likely at least partly genetic (41). This means that breeders are advised to formulate a breeding strategy to reduce the frequency of IE in the Labradoodle. Continued recording and monitoring of epilepsy in the Labradoodle may further our understanding of its genetic architecture.

## Conclusion

IE and EUC does occur in the Labradoodle. The semiology is comparable with most breeds. AoO is lower compared to several other breeds, but the outcome is good in most dogs. A large number of dogs did not receive an ASM or only one. On average, seizure frequency, compared to other breeds, was low. The seizure frequency and need for an ASM are comparable with Standard Poodles. The only clear difference with the Standard Poodles is the existence of CS and SE in the Labradoodle which is rarely seen in the Standard Poodles. QoL scores were high for both dog and owner.

## Data Availability

The raw data supporting the conclusions of this article will be made available by the authors, without undue reservation.

## Footnotes

^1^https://wagwalking.com/breed/top-designer-dog-breeds

^2^ALAEU – Australian Labradoodle Association Europe: https://alaeu.com/.

^3^WALA – Worldwide Australian Labradoodle Association: https://www.wala-labradoodles.org/.

^4^Qualtrics: https://www.qualtrics.com/.
