# Supplementary material for: Phenotypic characterization and quality of life of Labradoodles with idiopathic epilepsy and epilepsy of unknown cause
Source: Front Vet Sci. 2024 Oct 16;11:1459260. doi: 10.3389/fvets.2024.1459260 (PMC11523295; doi:10.3389/fvets.2024.1459260)
Supplement: Supplementary file 1 [file Data_Sheet_1.pdf]

Epilepsie onderzoek bij de Labradoodle

Start van blok: intro

Q1
Beste eigenaar,
Hartelijk dank voor uw bijdrage. Deze is zeer waardevol. Helaas komt epilepsie bij de Labradoodle vaak voor en is soms erg slecht te behandelen. De aandoening heeft dan ook een behoorlijke impact op het leven van de hond en u als eigenaar. Daarom zijn we bezig met dit onderzoek. Deels gaat het om het in kaart brengen van de epilepsie, hoe we het beter kunnen behandelen en of we ook een genetische oorzaak kunnen vinden. Meer informatie over epilepsie is gepubliceerd op de website www.veterinair-neuroloog.nl Er zijn diverse varianten 'doodles'. Dit kunt u in de enquête aangeven.

Genetisch onderzoek is alleen mogelijk als we ook DNA van uw hond krijgen. Een formulier waarmee u dit kunt inzenden kunt u hier downloaden. Mocht uw hond reeds overleden zijn is er geen DNA beschikbaar maar uw bijdrage blijft waardevol. Indien u ons financieel wilt ondersteunen dan is ook dat van harte welkom. Klik dan op deze link. Dit onderzoek is een project van de faculteit Diergeneeskunde in Utrecht (Nederland) en is onderdeel van lopend onderzoek van het Expertise Centrum Genetica van Gezelschapsdieren. Hierbij werken we samen met het Epicentrum van de faculteit Diergeneeskunde in Merelbeke (Gent-België). Indien wij nog geen bloedmonster van uw hond hebben, en het is mogelijk dan ontvangen wij dit graag! Het verzoek is dan uw dierenarts te vragen 4 ml EDTA bloed (niet gestold) naar ons op te sturen. Indien uw het formulier niet heeft dan kunt u ook downloaden via de volgende website: https://www.veterinair-neuroloog.nl/onderzoek/epilepsie-onderzoek-bij-de-labradoodle Het invullen van de enquête duurt 30 minuten. Dat is lang maar wij denken dat de antwoorden ons echt verder gaan helpen. Voordat u verder gaat kunt u het beste de stamboom, het vaccinatie boekje, epilepsie dagboek en de medicatie van uw hond erbij pakken. Niet bij iedere vraag is een antwoord vereist. Bij sommige wel. Alle gegevens worden vertrouwelijk verwerkt en worden niet gedeeld met derden. Wij hanteren dus de regels inzake de AVG. Indien u vragen en/of opmerkingen heeft dan kunt u deze op de laatste pagina van de enquête plaatsen.

Misschien twijfelt u of uw hond epilepsie heeft. Kijk dan vooraf naar deze filmpjes.

Zowel focale als gegeneraliseerde aanvallen zijn mogelijk. Indien u eerst meer hierover wilt lezen dan kunt u naar deze website gaan voor meer informatie: https://www.veterinair-neuroloog.nl/ziektes/epilepsie

Voorbeelden van focale aanvallen kunt u hier zien: https://youtu.be/AZhL9-_MvEM

Een voorbeeld van een zogenaamde tonisch-clonische aanval kunt u hier zien: https://youtu.be/6ticKGNgojw

Naast dit komt bij de labradoodle ook een zogenaamde parxosymale dyskinesie voor. Een voorbeeld hiervan kunt u hier zien: https://youtu.be/96UMxPZH1OA

Het kan zijn dat u beide types bij uw hond ziet. Als er twijfel is neem dan contact met ons op.   Hartelijke dank voor het invullen van de enquête.
dr. Paul Mandigers (Veterinaire Neurologie, Universiteit Utrecht & Evidensia Dierenziekenhuis Arnhem)

Q114 Gaat u akkoord met het gebruik van uw ingevulde gegevens voor onderzoek?

○ Akkoord (1)

○ Niet akkoord (2)

Deze vraag weergeven:

If Gaat u akkoord met het gebruik van uw ingevulde gegevens voor onderzoek? = Niet akkoord

Q115 U bent niet akkoord gegaan met het gebruik van uw ingevulde gegevens, hiermee heeft u de einde van deze enquête bereikt.

Ga naar: Einde enquête Als U bent niet akkoord gegaan met het gebruik van uw ingevulde gegevens, hiermee heeft u de einde va... wordt weergegeven

Einde blok: intro

Start van blok: Informatie eigenaar

Q8 Informatie over uzelf. Invoeren is niet verplicht maar wij denken dat een belangrijke factor de interactie tussen hond en eigenaar is. Vandaar deze vragen.

Q5 Woonplaats? (invoeren niet verplicht)

____

Q6 Wat is uw emailadres? (Invoeren is niet verplicht maar wel gewenst zodat we u bij onduidelijkheden achteraf kunnen bereiken).

____

Q116
Hoe omschrijft u uw eigen levenssituatie. Er volgen meerdere vragen. Doorloop ze AUB allemaal.

Ik ben,...

- ○ Alleenstaand (1)
- ○ Leef samen met mijn partner (2)
- ○ Een gezin met kinderen (3)

Q117 Waar woont u?

- ○ Ik woon landelijk / in een dorp en het is hier rustig (1)
- ○ Ik woon landelijk / in een dorp maar er gebeurd wel eens was (2)
- ○ Ik woon in een drukke omgeving maar het is geen stad (3)
- ○ Ik woon in een stad maar het is hier rustig (4)
- ○ Ik woon in een stad en het is hier altijd druk (5)
- ○ Anders, ik omschrijf het zelf wel (6) ____

Q122 Uw huis situatie:

- ○ Het is thuis altijd rustig (1)
- ○ Er is thuis altijd wel wat te doen maar het is niet druk (2)
- ○ Het is altijd wel druk thuis (3)
- ○ Het is thuis enorm druk en er is altijd veel bezigheid (4)
- ○ Anders, ik omschrijf het zelf wel (5) ________________

Q118 Ik omschrijf mijzelf als....

- ○ Een zeer rustig evenwichtig persoon (1)
- ○ Een bezig maar evenwichtig persoon (2)
- ○ Een erg druk maar evenwichtig persoon (3)
- ○ Een extreem druk en soms wel gehaast persoon (4)
- ○ Nogal stressvol bestaan (5)
- ○ Extreem stressvol (6)
- ○ Anders, ik omschrijf het zelf wel (7) ________________

Q120 Indien u samen leeft met anderen (een partner of met kinderen). Indien niet van toepassing sla deze vraag dan over.

- ○ Mijn huisgenoten zijn rustig en evenwichtige personen (1)
- ○ Mijn huisgenoten zijn bezig maar evenwichtige personen (2)
- ○ Mijn huisgenoten zijn erg druk maar nog steeds evenwichtige personen (3)
- ○ Mijn huisgenoten zijn extreem druk en soms ook wel erg gehaast (4)
- ○ Mijn huisgenoten maken het een nogal stressvol bestaan (5)
- ○ Mijn huisgenoten maken het extreem stressvol (6)
- ○ Anders, ik omschrijf het zelf wel (7) ____

Q121 Indien u meerdere huisdieren heeft: wat is dan van toepassing. Indien niet van toepassing sla deze vraag dan over.

- ○ Ik heb nog een of meerdere honden (1)
- ○ Ik heb ook nog een of meerdere katten (2)
- ○ Ik heb zowel een of meerdere katten en honden (3)
- ○ Ik heb geen andere huisdieren (4)
- ○ Anders, ik omschrijf het zelf wel (8) ____

Q123 Indien u meerdere huisdieren heeft: wat is dan van toepassing. Indien niet van toepassing sla deze vraag dan over.

- ○ Er is geen gedoe tussen mijn verschillende huisdieren (1)
- ○ Er is soms wat gedoe tussen mijn verschillende huisdieren (2)
- ○ Er is constant gedoe tussen mijn verschillende huisdieren (3)
- ○ Ik heb geen andere huisdieren (4)
- ○ Anders, ik omschrijf het zelf wel (8) ____

Einde blok: Informatie eigenaar

Start van blok: Vragen hond, vaccinatie etc

Q9 Vragen over uw hond, vaccinatie etc voorgeschiedenis.

Q17 Roepnaam van uw hond?

____

Q18 Heeft uw hond een stamboom van de Australian Labradoodle Club (ALAEU) of van de Dutch Australian Labradoodle club (DALC)?

- ▢ Ja, van de ALAEU (1)
- ▢ Ja, van de DALC (2)
- ▢ Het is een labradoodle en hij heeft een registratie maar geen van bovenstaande, ik vul het in (3)
- ▢ Het is een labradoodle maar hij heeft geen registratienummer (4)
- ▢ Anders. DWZ het is een doodle maar ik vul in wat het is (5) ____
- ▢ Het is geen doodle maar anders, ik vul het in (6) ____

Q19 Indien van toepassing: wat is de stamboeknaam van uw hond?

____

Q20 Indien van toepassing:
Stamboomnummer (graag volledig nummer inclusief cijfers van de uitgever. Dus bijvoorbeeld ALAEU 000000)

____

Q21 Chipnummer van uw hond?

____

Q22 Wat is de geboortedatum van uw hond? U mag het opschrijven of zo invullen: dd/mm/jjjj

____

____

____

____

____

Q23 Wat is het geslacht van uw hond?

- ○ Mannelijk (1)
- ○ Vrouwelijk (2)

Q24 Is uw hond gesteriliseerd of gecastreerd?

- ○ Ja (1)
- ○ Nee (2)

Q25 Indien gesteriliseerd of gecastreerd: weet u nog bij benadering de datum waarop de sterilisatie/castratie plaats heeft gevonden?(dd/mm/jjjj)

____

____

____

____

____

Pagina-einde

Q27 Werd uw hond gevaccineerd voordat hij/zij de eerste epileptiforme aanval kreeg?

- ☐ Ja (1)
- ☐ Nee (2)
- ☐ Indien het antwoord JA is. Welk merk? Dit kunt u in het vaccinatieboekje terug vinden. (3) ____________________
- ☐ Indien het antwoord JA is. Wanneer was dat (4) ____________________

Q28 Kreeg uw hond ontwormingstabletten voordat hij/zij de eerste epileptiforme aanval kreeg?

- ☐ Ja (1)
- ☐ Nee (2)
- ☐ Indien JA: weet u ook wat het merk van deze ontworming was? (3) ____________________
- ☐ Indien JA: weet u ook wanneer dat ongeveer was? (4) ____________________

Q29 Heeft u uw hond behandeld met anti-vlooien/teken producten voordat hij/zij de eerste epileptiforme aanval kreeg?

- ☐ Ja (1)
- ☐ Nee (2)
- ☐ Indien JA: welke medicatie was dit? (3) ____
- ☐ Indien JA: weet u ook wanneer dat ongeveer was? (4) ____

Q30 Wat was de voeding die uw hond kreeg voordat hij/zij de eerste epileptiforme aanval kreeg (Indien mogelijk merk vermelden)?

- ○ Type voeding 1 (1) ____
- ○ Type voeding 2 (2) ____
- ○ Type voeding 3 (3) ____

Q31 Is de voeding die hij/zij nu krijgt veranderd?

- ○ Ja (1)
- ○ Nee (2)
- ○ Indien ja, wat krijgt hij/zij nu? (3) ____

Q32 Heeft uw hond, voordat hij/zij de eerste epileptiforme aanval kreeg, een serieuze ziekte gehad? Indien u twijfelt vul dan altijd Ja in en vermeld welke ziekte het was.

- ☐ Ja (1)
- ☐ Nee (2)
- ☐ Indien JA: vul dan hier in wat voor een ziekte/ziektes het was. (3) ____

Einde blok: Vragen hond, vaccinatie etc

Start van blok: Woonsituatie

Q10 Algemene vragen over uw hond.

Q35 Hoe omschrijft u het karakter van uw hond? (meerdere antwoorden zijn mogelijk)

- ☐ Levendig (1)
- ☐ Opgewekt (2)
- ☐ Kalm (3)
- ☐ Zenuwachtig (4)
- ☐ Angstig (5)
- ☐ Agressief (6)
- ☐ Overige (geef nadere toelichting) (7) ____

Q36 Leeft uw hond grotendeels binnen of buitenhuis?

- ☐ Binnenhuis (1)
- ☐ Buitenhuis (2)
- ☐ Een combinatie van beide (3)
- ☐ Overige (geef toelichting) (4) ____

Q37 Hoe veel uren per dag is uw hond onder uw, of van een van uw huisgenoten, toezicht?

- ○ Minder dan 5 uur per dag (1)
- ○ Vijf tot 10 uur per dag (2)
- ○ Tien tot 15 uur per dag (3)
- ○ Vijftien tot 20 uur per dag (4)
- ○ Meer dan 20 uur per dag (5)

Einde blok: Woonsituatie

Start van blok: Eerste aanvallen

Q11 Vragen over de (eerste) aanvallen.

Q126 Wanneer heeft u de eerste aanval bij uw hond gezien? Indien u de datum niet exact meer weet, geef dan een datum bij benadering. (dd/mm/jjjj)

____

____

____

Q127 Wanneer is de meest recente aanval opgetreden?

____

____

____

____

____

Q40 Heeft uw hond meer dan 1 aanval gehad?

○ Ja (1)

○ Nee (2)

*

Q41 Hoeveel aanvallen heeft uw hond tot op heden gehad? Vul een getal in.

____

Q44
Honden kunnen diverse soorten epileptiforme aanvallen krijgen. Zowel focale als gegeneraliseerde aanvallen zijn mogelijk. Indien u eerst meer hierover wilt lezen dan kunt u naar deze website gaan voor meer informatie: https://www.veterinair-neuroloog.nl/ziektes/epilepsie

Voorbeelden van focale aanvallen kunt u hier zien: https://youtu.be/AZhL9-_MvEM

Een voorbeeld van een zogenaamde tonisch-clonische aanval kunt u hier zien: https://youtu.be/6ticKGNgojw

Naast dit komt bij de labradoodle ook een zogenaamde parxosymale dyskinesie voor. Een voorbeeld hiervan kunt u hier zien: https://youtu.be/96UMxPZH1OA

Het kan zijn dat u beide types bij uw hond ziet. Als er twijfel is neem dan contact met ons op.

Q124 Wat voor type aanval(len) vertoont uw hond?

- ☐ Gegeneraliseerde tonisch-clonische aanvallen. Dit zijn aanvallen waarbij de hond in de regel op zijn zijde ligt, krampen en fietsbewegingen heeft, trekkingen met de mond, in de regel krijgt u geen contact met hem/haar en kan speeksel, urine en ontlastingsverlies optreden. (1)
- ☐ Focale aanvallen. Dit zijn, bijvoorbeeld, aanvallen waarbij slechts een deel van het lichaam de abnormaliteit laat zien. Voorbeelden zijn: trekken met de lippen, speekselen, alleen een pootje etc. (2)
- ☐ Een combinatie van zowel tonisch-clonische aanvallen en de focale aanvallen. (3)
- ☐ Overige, namelijk... (4) ____

Q42 Weet u wat de oorzaak van de aanvallen bij uw hond is? Slechts een antwoord is mogelijk

- ○ Idiopathisch (dit wil zeggen: we weten de oorzaak niet, vaak vermoedelijk genetisch). Maar er is geen oorzaak gevonden. (2)
- ○ Metabool / toxisch / reactief (= de oorzaak ligt buiten de hersenen. Denk aan bijv een lever, nier of suikertekort). Er is dan bloedonderzoek uitgevoerd waaruit bleek dat de oorzaak dus niet idiopathisch is. (3)
- ○ Structureel of secundair (= de oorzaak zit in de hersenen. Denk aan bijv trauma , ontsteking, tumor) (4)
- ○ Overige (geef nadere toelichting) (5) ____

Q43 Wie heeft deze diagnose gesteld?

- ☐ Ikzelf (1)
- ☐ Mijn dierenarts (2)
- ☐ De specialist (3)
- ☐ Overige (geef nadere toelichting) (4) ____

Q45 Wie is de behandelaar van de epilepsie bij uw hond?

- ◯ (Eerstelijns) dierenarts (1)
- ◯ Specialist veterinaire neurologie (2)
- ◯ Specialist veterinaire neurologie in opleiding (SIO) (3)
- ◯ Specialist interne geneeskunde (4)
- ◯ Overige (geef nadere toelichting) (5) ____

Deze vraag weergeven:

If Wie is de behandelaar van de epilepsie bij uw hond? = Specialist veterinaire neurologie

Or Wie is de behandelaar van de epilepsie bij uw hond? = Specialist veterinaire neurologie in opleiding (SIO)

Or Wie is de behandelaar van de epilepsie bij uw hond? = Specialist interne geneeskunde

Or Of Wie is de behandelaar van de epilepsie bij uw hond? Overige (geef nadere toelichting) Bevat specialist

Q46 Hoe bent u terecht gekomen bij de specialist?

- ◯ Dit heb ik zelf geregeld (1)
- ◯ Dit heb ik gedaan op advies van mijn dierenarts (2)
- ◯ Anders, namelijk... (3) ____

Einde blok: Eerste aanvallen

Start van blok: Aanvullende vragen

Q12 Aanvullende vragen over de aanvallen.

Q47 Is uw hond nog in leven?

◯ Ja (1)

◯ Nee (2)

Deze vraag weergeven:

If Is uw hond nog in leven? = Nee

Q48 Op welke leeftijd is uw hond overleden? (in jaartallen)

____

Deze vraag weergeven:

If Is uw hond nog in leven? = Nee

Q49

Wat was de oorzaak van het overlijden van uw hond?

◯ Ten gevolge van het feit dat hij epilepsie had (1)

◯ Anders, namelijk (3) ____

Pagina-einde

Q50 Hoeveel aanvallen had uw hond in het EERSTE jaar nadat de eerste aanval is gezien?

- ○ Aantal aanvallen per jaar (1) ________________
- ○ Aantal aanvallen de eerste zes maanden (2) ________________
- ○ Aantal aanvallen de eerste drie maanden (3) ________________
- ○ Aantal aanvallen de eerste maand (indien van toepassing) (4) ________________
- ○ Aantal aanvallen de eerste week (indien van toepassing) (5) ________________

Q51 Hoeveel aanvallen heeft uw hond dit LAATSTE jaar gehad?

- ○ Niet van toepassing: dit is pas het eerste jaar (1) ________________
- ○ Aantal aanvallen het laatste jaar (2) ________________
- ○ Aantal aanvallen de laatste zes maanden (3) ________________
- ○ Aantal aanvallen de laatste drie maanden (4) ________________
- ○ Aantal aanvallen de laatste maand (indien van toepassing) (5) ________________
- ○ Klik om formulierveld 6 te schrijven (6) ________________

Q52 Hoe beoordeelt u de ernst van de aanvallen NU ten opzichte van de eerste aanvallen. U kunt nu met de schuifbalk aangeven of het minder of erger is geworden.

Indien u de balk in het midden zet is het gelijk gebleven. Naar links minder en naar rechts meer. Nul betekent niet ernstig, Tien betekent heel ernstig.

	0	1	2	3	4	5	6	7	8	9	10
0=niet ernstig, 5=gelijk gebleven, 10=heel ernstig ()											

Q53 Hoe lang heeft het geduurd voordat u met een anti-epilepticum bent gestart: gerekend vanaf de eerste aanval die u heeft gezien.

○ We zijn gelijk, de eerste week, na de eerste aanval begonnen met een medicatie. (1)

○ We zijn nog niet begonnen met een medicatie. (2)

○ We hebben gewacht: vul hier het aantal maanden tussen de eerste aanval en de start van de medicatie in. (3) ____________________

Q54 Kunt u een trigger (aanleiding) aanwijzen voor het optreden van de aanvallen? Meerdere antwoorden zijn mogelijk.

- ☐ Stress (1)
- ☐ Sexuele opwinding (2)
- ☐ Weersinvloeden (3)
- ☐ Bezoek aan huis (4)
- ☐ Bezoek aan de dierenarts (5)
- ☐ Er is een duidelijk tijdstip van de dag aanwezig (6)
- ☐ Er is een duidelijke seizoensinvloed aanwezig (7)
- ☐ Ik herken geen trigger (10)
- ☐ Anders, ik vul het zelf in (11) ____

Pagina-einde

Q55 U gaf aan dat uw hond gesteriliseerd of gecastreerd is. Is dit uitgevoerd NADAT de hond aanvallen heeft ontwikkeld?

- ◯ Ja, na ontwikkeling aanvallen (1)
- ◯ Nee, voor ontwikkeling aanvallen (2)
- ◯ Weet ik niet meer (3)

Deze vraag weergeven:

If U gaf aan dat uw hond gesteriliseerd of gecastreerd is. Is dit uitgevoerd NADAT de hond aanvallen... = Ja, na ontwikkeling aanvallen

Q56 Is de aanvalsfrequentie verandert sinds de sterilisatie / castratie?

- ◯ De aanvallen namen in frequentie af. (1)
- ◯ De aanvalsfrequentie veranderde niet. (2)
- ◯ De aanvalsfrequentie nam toe (3)
- ◯ Overige (geef nadere toelichting) (4) ____

Q57 Is uw hond verwant aan andere epilepsielijders?

- ◯ Ja (1)
- ◯ Nee (2)
- ◯ Weet ik niet (3)
- ◯ Indien ja, wat is de verwantschap? (4) ____

Pagina-einde

Q58 Vaak zien we een inleidende fase. Dat is niet de aanval zelf maar een fase (seconden tot dagen voor de werkelijke aanval) voor de aanval. Herkent u deze bij uw hond?

- ○ Ja (1)
- ○ Nee (2)
- ○ Weet ik niet (3)

Q59 Indien u een inleidende fase ziet: kunt u deze dan beschrijven?

- ○ Misselijkheid (1)
- ○ Braken (2)
- ○ Speekselen / kwijlen (3)
- ○ Rusteloosheid (4)
- ○ Hij/Zij zoekt contact met mij/ons (5)
- ○ De hond wordt agressief (6)
- ○ Overige (geef nadere toelijchting) (7) ____

Deze vraag weergeven:

If Vaak zien we een inleidende fase. Dat is niet de aanval zelf maar een fase (seconden tot dagen vo... = Ja

Q60 Hoe lang voordat de aanval optreedt ziet u deze inleidende fase? Geef het svp weer in minuten.

____

Q61 Kunt u het optreden van een aanval voorspellen?

- ◯ Ja, altijd (100% van de keren) (1)
- ◯ Ja, in 75% van de keren (2)
- ◯ Ja, in 50% van de keren (3)
- ◯ Ja, in 25% van de keren (4)
- ◯ Nee (5)

Q62 Heeft u ooit gezien dat uw hond zich bewust was van een aanval?

- ◯ Ja (1)
- ◯ Nee (2)
- ◯ Overige (geef nadere toelichting) (3) ____

Q63 Wat doet uw hond veelal voordat een aanval optreedt?

- ◯ Hij/zij sliep (1)
- ◯ Hij/zij werd net wakker (2)
- ◯ Hij/zij was wakker maar lag in zijn mand/kleed te rusten (3)
- ◯ Liep buiten (4)
- ◯ Was aan het spelen (5)
- ◯ Was aan het sporten (6)
- ◯ Ik zie het nooit (7)
- ◯ Overige (geef nadere toelichting) (8) ____

Q64 Kunt u contact met uw hond maken tijdens de aanval?

- ◯ Ja (1)
- ◯ Nee (2)
- ◯ Weet ik niet (3)

Deze vraag weergeven:

If Kunt u contact met uw hond maken tijdens de aanval? = Ja

Q65 Indien u contact maakt tijdens een aanval. Hoe reageert de hond dan?

- ○ Volledig bij, reageert gewoon (1)
- ○ Is anders maar lijkt wel te reageren (2)
- ○ Reageert totaal niet op mij (3)
- ○ Anders, ik vul het zelf in (4) ________________

Pagina-einde

Q66 Hoe lang duren de aanvallen gemiddeld? Vul minuten in. Vul hier niet de tijd voor de aanval (inleidende fase) of na de aanval (post-ictale fase) in. Puur alleen de aanval.

____

Q69

Hoe lang duurde de kortste aanval? Vul minuten in.

____

*

Q68

Hoe lang duurde de langste aanval? Vul minuten in.

____

Einde blok: Aanvullende vragen

Start van blok: Vragen over de aanvallen zelf

Q13 Vragen over de aanvallen zelf

Q70 Hieronder staan meerdere zaken genoemd die bij een aanval kunnen optreden. Wilt u dat wat u regelmatig ziet aanklikken? Meerdere antwoorden zijn mogelijk.

- ▢ Verkrampen van de poten en de nek (1)
- ▢ Neervallen (2)
- ▢ Tijdens de aanval ligt de hond op een zijde (3)
- ▢ Fiets bewegingen met de poten (4)
- ▢ Draaien van het hoofd (5)
- ▢ Kauwbewegingen met de bek (6)
- ▢ Trekkingen van de aangezichtsspieren (7)
- ▢ Urineverlies (8)
- ▢ Ontlastingsverlies (9)
- ▢ Speekselen (10)
- ▢ Verwijding van de pupillen (11)
- ▢ Rondjes lopen (12)
- ▢ Staartjagen (13)
- ▢ Bewustzijnsverlies (14)
- ▢ Staren (15)
- ▢ Contact zoeken met mensen (16)
- ▢ Tegen meubels / andere zaken oplopen (17)

- [ ] Ziet slecht / tijdelijk zichtverlies (18)
- [ ] Blaffen (19)
- [ ] Angst (20)
- [ ] Agressie (21)
- [ ] Overig (geef nadere toelichting) (22) ____

Q71 Zien alle aanvallen er steeds hetzelfde uit?

- [ ] Ja (1)
- [ ] Nee (2)
- [ ] Indien u nee antwoordde. Kunt u dit aangeven waarom niet? (3) ____

Q72 Is uw hond tussen de aanvallen door normaal? Dus in de periodes dat er geen aanvallen zijn?

- ○ Ja het is verder een normale en leuke Labradoodle (1)
- ○ Nee (2)
- ○ Weet ik niet (3)
- ○ Indien u het niet weet of nee als antwoord geeft kunt u dit dan verduidelijken? (4) ____

Q73 Heeft u de indruk dat de linker of juist de rechterhelft van het lichaam tijdens een aanval meer actief is (denk bijvoorbeeld aan het steeds naar een zijde bewegen van het hoofd)?

- ○ Ja er is een verschil (of het links of rechts is maakt voor dit antwoord niet uit) (1)
- ○ Nee ik zie geen links-rechts verschil (2)
- ○ Weet ik niet (3)

Q74 Kunt u de aanval verkorten?

- ▢ Ja (1)
- ▢ Nee (2)
- ▢ Weet ik niet (3)
- ▢ Indien ja, wat doet u dan? (4) ____

Einde blok: Vragen over de aanvallen zelf

Start van blok: Post-ictale fase

Q14 Vragen over de fase na de aanval: de post-ictale fase. Deze fase kan seconden tot dagen duren. **Vaak lopen ze dan doelloos rond, zijn afwezig, zien vaak slecht en zo verder,..**

Q75 De post-ictale fase is de fase na de aanval. Deze fase is soms niet herkenbaar, soms juist dagen aanwezig. Heeft uw hond een post-ictale fase?

- ○ Ja (1)
- ○ Nee (2)
- ○ Weet ik niet (3)

Q76 Heeft u de indruk dat uw hond zich de aanval herinnert?

- ◯ Ja (1)
- ◯ Nee (2)
- ◯ Weet ik niet (3)

Q77 Hoe lang duurt het voordat de post-ictale fase voorbij is? Geef het weer in minuten.

____

Q78 Reageert de hond op u tijdens de post-ictale fase?

- ◯ Ja (1)
- ◯ Nee (2)
- ◯ Weet ik niet (3)

Q79 Wat ziet u tijdens de post-ictale fase?

- ☐ De hond is moe (1)
- ☐ De hond loopt doelloos rond (2)
- ☐ De hond lijkt blind te zijn (3)
- ☐ De hond is agressief (4)
- ☐ De hond wil gelijk drinken (5)
- ☐ De hond wil gelijk eten (6)
- ☐ De hond wil wandelen (7)
- ☐ De hond wil niets doen en blijft liggen (8)
- ☐ De hond braakt (9)
- ☐ De hond gaat zich uitrekken (10)
- ☐ Overige (geen nadere toelichting) (11)

Einde blok: Post-ictale fase

Start van blok: Dierenarts

Q15 Wat uw dierenarts zoal heeft gedaan?

Q80 Wat heeft uw dierenarts zoal gedaan om te komen tot de diagnose?

- ☐ Klinisch onderzoek (1)
- ☐ Hartecho (2)
- ☐ Bloedonderzoek (3)
- ☐ ECG (hartfilmpje) (4)
- ☐ ECHO hart (5)
- ☐ EEG (Electro-encephalogram - hersenonderzoek) (6)
- ☐ MRI scan van de hersenen (7)
- ☐ CT scan van de hersenen (8)
- ☐ Hersenvochtonderzoek: CSF / CSV onderzoek / liquor punctie (9)
- ☐ Overige (geef nadere toelichting) (10)
- ☐ Urineonderzoek (11)

Q81 Heeft uw hond, naast de epilepsie, ook andere problemen?

- ☐ Ja (1)
- ☐ Nee (2)
- ☐ Indien Ja, geef hier aan welke (3)

____

Einde blok: Dierenarts

Start van blok: Geslacht specifiek

Q16 Enkele specifieke vragen over reuen en teven

Deze vraag weergeven:

If Wat is het geslacht van uw hond? = Vrouwelijk

Q82 Op welke leeftijd is uw teefje voor het eerst loops geworden?

- ☐ Ze is nog niet loops geweest (1)
- ☐ Niet van toepassing voor mijn hond (2)
- ☐ Haar eerste loopsheid zagen we: (3) ________________

Deze vraag weergeven:

If Wat is het geslacht van uw hond? = Vrouwelijk

Q83 Is de loopsheid regelmatig?

- ○ Ja (1)
- ○ Nee (2)
- ○ Weet ik niet (3)
- ○ Niet van toepassing (4)

Deze vraag weergeven:

If Wat is het geslacht van uw hond? = Vrouwelijk

Q84 Heeft uw teef ooit pups gehad?

- ▢ Ja (1)
- ▢ Nee (2)
- ▢ Weet ik niet (3)
- ▢ Indien Ja, hoeveel nesten? (4) ________________

Deze vraag weergeven:

If Wat is het geslacht van uw hond? = Mannelijk

Q85 Voor reuen: Heeft uw hond een normaal sexueel gedrag?

- ▢ Ja (1)
- ▢ Nee (2)
- ▢ Weet ik niet (3)
- ▢ Indien neen: wat is dan abnormaal? (4) ________________

Deze vraag weergeven:

If Wat is het geslacht van uw hond? = Mannelijk

Q86 Voor reuen: heeft uw hond nakomelingen?

- ☐ Ja (1)
- ☐ Nee (2)
- ☐ Weet ik niet (3)
- ☐ Indien Ja, hoeveel nesten? (4) ____

Einde blok: Geslacht specifiek

Start van blok: Aantallen, ernst, controle

Q87 Hoeveel aanvallen heeft uw hond de laatste maanden gehad? Tel hierbij de clusters* als een aanval. *Met een cluster wordt bedoeld: aanvallen die op elkaar volgen (binnen 24 uur) waarbij de hond tussen de aanvallen door een normaal bewustzijn heeft.

- ○ De laatste 12 maanden heeft mijn hond ... aanvallen gehad (1) ____
- ○ De laatste 6 maanden heeft mijn hond ... aanvallen gehad (2) ____
- ○ De laatste 3 maanden heeft mijn hond ... aanvallen gehad (3) ____
- ○ De laatste maand heeft mijn hond ... aanvallen gehad (4) ____

Q88 Hoeveel clusteraanvallen* heeft uw hond de laatste maanden gehad?Vul hier alleen de clusters in. Indien u ze niet ziet vult u hier dus niets in. * Met clusteraanvallen wordt bedoeld: aanvallen die

op elkaar volgen (binnen 24 uur) maar waarbij de hond tussen de aanvallen door een normaal bewustzijn heeft.

- ○ De laatste 12 maanden heeft mijn hond ... clusteraanvallen gehad (1) ________________
- ○ De laatste 6 maanden heeft mijn hond ... clusteraanvallen gehad (2) ________________
- ○ De laatste 3 maanden heeft mijn hond ... clusteraanvallen gehad (3) ________________
- ○ De laatste maand heeft mijn hond ... clusteraanvallen gehad (4) ________________

Q89 Hoe vaak is er in de laatste maanden sprake geweest van een status epilepticus*? * Status epilepticus houdt in dat de toevallen elkaar binnen zeer korte tijd opvolgen, waarbij er geen herstelfase is

- ○ De laatste 12 maanden is er ... keer sprake geweest van een status epilepticus (1) ________________
- ○ De laatste 6 maanden is er ... keer sprake geweest van een status epilepticus (2) ________________
- ○ De laatste 3 maanden is er ... keer sprake geweest van een status epilepticus (3) ________________
- ○ De laatste maand is er ... keer sprake geweest van een status epilepticus (4) ________________

Pagina-einde

Q90 De ernst van de tonisch clonische aanvallen beoordeel ik als: (met 0 niet ernstig en 10 zeer ernstig)

Sleep de schuifregelaar naar de gewenste positie

	0	1	2	3	4	5	6	7	8	9	10
0=niet ernstig, 10=zeer ernstig ()											

Q92 Indien aanwezig: De ernst van de focale aanvallen beoordeel ik als: (met 0 niet ernstig en 10 zeer ernstig)

Sleep de schuifregelaar naar de gewenste positie

	0	1	2	3	4	5	6	7	8	9	10
0=niet ernstig, 10=zeer ernstig ()											

Q91 Aan het gedrag van de hond kan ik voorspellen wanneer er een aanval komt; mijn hond vertoont een veranderd gedrag enige tijd voor de aanval.
(met 0 niet eens en 10 zeer eens) Sleep de schuifregelaar naar de gewenste positie

	0	1	2	3	4	5	6	7	8	9	10
0=niet eens, 10=zeer eens ()											

Pagina-einde

Q93 Hoe vaak komt uw hond op controle bij een dierenarts of specialist?

- ○ Eens per week (1)
- ○ Eens per 2 weken (2)
- ○ Eens per 3 weken (3)
- ○ Eens per maand (4)
- ○ Eens per 2 maanden (5)
- ○ Eens per 3 maanden (6)
- ○ Eens per 4 maanden (7)
- ○ Eens per 5 maanden (8)
- ○ Eens per 6 maanden (9)
- ○ Eens per jaar (10)
- ○ Nooit (11)
- ○ Anders, namelijk.. (12)

Q94 Hoe vaak moest u in de afgelopen maanden in de avonduren of in het weekend naar een dierenarts in verband met de epilepsie van uw hond?

- ○ De afgelopen 12 maanden ... keer (1) ________________
- ○ De afgelopen 6 maanden ... keer (2) ________________
- ○ De afgelopen 3 maanden ... keer (3) ________________
- ○ De afgelopen maand ... keer (4) ________________

Einde blok: Aantallen, ernst, controle

Start van blok: Medicatie

Q95 Welke medicatie, in tablet of capsule vorm, gebruikt uw hond? (meerdere antwoorden mogelijk)

- ▢ Fenobarbital (merknamen: Phenoral, Phenoleptil en Soliphen) (1)
- ▢ Imepitoine (Pexion) (3)
- ▢ Kaliumbromide (merknamen: Epikal, Libromide) (4)
- ▢ Gabapentine (merknamen: Neurontin of Gabapentine) (7)
- ▢ Levetiracitam (merknamen: Keppra of levetiracetam) (8)
- ▢ Geen medicatie (9)
- ▢ Anders, namelijk... (10) ________________

Q125 Welke extra medicatie (vaak nood) gebruikt uw hond? (meerdere antwoorden mogelijk)

- ▢ Neusspray midazolam (5)
- ▢ Diazepam, valium, stesolid (rectaal) (6)
- ▢ Levetiracitam (merknamen: Keppra of levetiracetam) (8)
- ▢ Gabapentine (merknamen: Neurontin of Gabapentine) (7)
- ▢ Geen medicatie (9)
- ▢ Anders, namelijk... (10) ____

Deze vraag weergeven:

If Welke medicatie, in tablet of capsule vorm, gebruikt uw hond? (meerdere antwoorden mogelijk) != Geen medicatie

And En Welke medicatie, in tablet of capsule vorm, gebruikt uw hond? (meerdere antwoorden mogelijk) q://QID95/SelectedChoicesCount is gelijk aan 1

Or Of Welke medicatie, in tablet of capsule vorm, gebruikt uw hond? (meerdere antwoorden mogelijk) q://QID95/SelectedChoicesCount is groter dan 1

Q96 Welke neveneffecten van de medicatie ziet u bij uw hond? (er zijn meerdere antwoorden mogelijk)

- ▢ Sloomheid (1)
- ▢ Meer slapen (2)
- ▢ Rusteloosheid (3)
- ▢ Prikkelbaar (4)
- ▢ Wankel zijn / ongecoördineerd (5)
- ▢ Spierzwakte / zwakte (6)
- ▢ Toename eetlust (7)
- ▢ Gewichtstoename (8)
- ▢ Braken (9)
- ▢ Diarree (10)
- ▢ In huis poepen (11)
- ▢ Meer drinken (12)
- ▢ Meer plassen (13)
- ▢ Hoesten (14)
- ▢ Huiduitslag (15)
- ▢ GEEN. Mijn hond heeft geen bijwerkingen (16)
- ▢ Anders, namelijk... (17) ____

Pagina-einde

Q97 Maakt uw hond gebruik van alternatieve behandelingsmethoden?

- Ja (1)
- Nee (2)

Deze vraag weergeven:

If Maakt uw hond gebruik van alternatieve behandelingsmethoden? = Ja

Q98 Van welke alternatieve behandelingsmethode(n) maakt uw hond gebruik? (meerdere antwoorden mogelijk)

- Fytotherapie (1)
- CBD olie (2)
- CBD/THC olie (3)
- MCT olie (4)
- Speciaal dieet (5)
- Muziektherapie (6)
- Anders, namelijk... (7) ____

Einde blok: Medicatie

Start van blok: Scores

Q99 Terugkijkend naar de periode voordat uw hond epilepsie kreeg, als die periode 100% goed was. Hoe scoort u uw hond dan nu? Het cijfer 0 drukt uit dat u de situatie erg slecht vindt en 100 betekent dat het erg goed gaat.

Sleep de schuifregelaar naar de gewenste positie

0 10 20 30 40 50 60 70 80 90 100

0=het gaat erg slecht, 100=het gaat erg goed ()	

Q101 In de laatste 3 maanden heb ik mij zorgen gemaakt over de frequentie van de aanvallen bij mijn hond. Sleep de schuifregelaar naar de gewenste positie. Met 0 geen zorgen en 10 erg veel zorgen.

	0	1	2	3	4	5	6	7	8	9	10
0=geen zorgen, 10=erg veel zorgen ()											

Q102 De ernst van de aanvallen bij mijn hond is acceptabel voor mij.
Sleep de schuifregelaar naar de gewenste positie. Met 0 het gaat erg goed en 10 het gaat erg slecht

	0	1	2	3	4	5	6	7	8	9	10
0=het gaat erg goed, 10=het gaat erg slecht ()											

Q103 Ik durf mijn hond alleen thuis te laten. Sleep de schuifregelaar naar de gewenste positie. Met 0 geen enkel probleem en 10 ik durf hem/haar niet alleen te laten.

	0	1	2	3	4	5	6	7	8	9	10
0=geen enkel probleem, 10=ik durf hem/haar niet alleen te laten ()											

Q104 Het zorgen voor mijn hond met epilepsie beperkt mij in mijn dagelijkse bezigheden; het zorgt voor een afname van mijn eigen kwaliteit van leven.

Sleep de schuifregelaar naar de gewenste positie. Met 1 zegt u dat uw kwaliteit van leven op het dieptepunt zit. Met een 10 dat het geweldig gaat.

	1 2 3 4 5 6 6 7 8 9 10
1=een waar dieptepunt, 10=het gaat echt heel goed ()	

Q105 Het zorgen voor mijn hond met epilepsie is het waard.
Sleep de schuifregelaar naar de gewenste positie. Met 0 zegt u dat het echt een probleem is en met 10 dat u het echt de moeite waard vindt.

	0 1 2 3 4 5 6 7 8 9 10
0=een groot probleem, 10=geen enkel bezwaar ()	

Q106 Het toedienen van de medicatie bij mijn hond levert problemen op.
Sleep de schuifregelaar naar de gewenste positie. Met 0 zegt u dat het geen probleem is en met 10 dat het echt geen probleem is.

	0 1 2 3 4 5 6 7 8 9 10
0=geen probleem, 10=echt een probleem ()	

Q107 De neven-effecten van de medicatie bij mijn hond zijn acceptabel voor mij.
Sleep de schuifregelaar naar de gewenste positie. Met 0 geen probleem / geen bijwerkingen en 10 veel bijwerkingen: niet acceptabel.

	0 1 2 3 4 5 6 7 8 9 10
0=geen probleem, 10=veel bijwerkingen: niet acceptabel ()	

Q108 De kosten van de behandeling van epilepsie zijn acceptabel voor mij.
Sleep de schuifregelaar naar de gewenste positie. Met 0 niet acceptabel/echt een probleem en 10 zeer acceptabel

	0	1	2	3	4	5	6	7	8	9	10
0=niet acceptabel, 10=zeer acceptabel ()											

Q109 Is het voor u een probleem om bij de dierenarts of specialist op consult te gaan (voor bijvoorbeeld controles, onderzoek van de bloed concentraties van de medicatie)?
Sleep de schuifregelaar naar de gewenste positie. Met 0 geen enkel probleem en met 10 zegt u dat het een groot probleem is.

	0	1	2	3	4	5	6	7	8	9	10
0=geen probleem, 10=een zeer groot probleem ()											

Q110 Scoor de kwaliteit van leven van uw hond.
Sleep de schuifregelaar naar de gewenste positie. Met 1 zeer slecht en 10 uitstekend.

	1	2	3	4	5	6	6	7	8	9	10
1=zeer slecht, 10=uitstekend ()											

Pagina-einde

Q111 Welke fase van epilepsie vindt u de grootste aanslag op de kwaliteit van leven van uw hond?

- ◯ Inleidende fase / prodromale fase: Dit is een fase van abnormaal gedrag, mogelijk gepaard gaande met braken; het duurt enkele seconden tot dagen. (1)
- ◯ De aanval / ictus: Dit is een fase van bewustzijnsverlies en krampen; dit kan gepaard gaan met blaffen, kwijlen, plassen en poepen; dit duurt seconden tot minuten. (2)
- ◯ De hertselfase / post-ictale fase: Dit is een fase waarin de hond soms niet kan lopen, slaperig is, onrustig en mogelijk agressief is; het duurt seconden tot weken. (3)
- ◯ Geen specifieke fase (4)

Q112 Helaas is het erg moeilijk om volledige aanvalsvrijheid te verkrijgen. Maar wat vindt u redelijkerwijs acceptabel?

- ◯ Een aanval per week (1)
- ◯ Een aanval per 2 weken (2)
- ◯ Een aanval per 3 weken (3)
- ◯ Een aanval per maand (4)
- ◯ Een aanval per 2 maanden (5)
- ◯ Een aanval per 3 maanden (6)
- ◯ Een aanval per 4 maanden (7)
- ◯ Een aanval per 5 maanden (8)
- ◯ Een aanval per 6 maanden (9)
- ◯ Een aanval per jaar (10)
- ◯ Vrij van aanvallen (11)

Einde blok: Scores

Start van blok: Einde

Q113 Hartelijk bedankt voor uw medewerking aan dit onderzoek. Indien u interesse heeft in de resultaten van het onderzoek, dan kunt u hieronder uw e-mailadres doorgeven, dan zult u de uiteindelijke onderzoeksresultaten ontvangen.
Indien u vragen en/of opmerkingen heeft, kunt u die hier plaatsen.

____

____

____

____

____

Einde blok: Einde
